# Comparison of Effects of Different Statins on Contrast-Induced Acute Kidney Injury in Rats: Histopathological and Biochemical Findings

**DOI:** 10.1155/2017/6282486

**Published:** 2017-01-24

**Authors:** Xiao-lei Wang, Tuo Zhang, Liu-hua Hu, Shi-qun Sun, Wei-feng Zhang, Zhe Sun, Ling-hong Shen, Ben He

**Affiliations:** Department of Cardiology, Ren Ji Hospital, School of Medicine, Shanghai Jiao Tong University, Shanghai, China

## Abstract

Statins are a promising new strategy to prevent contrast-induced acute kidney injury (CI-AKI). In this study we compared the ameliorative effect of different statins in a rat model of CI-AKI. Sprague-Dawley rats were divided into five groups: control group; CI-AKI group; CI-AKI + rosuvastatin group (10 mg/kg/day); CI-AKI + simvastatin group (80 mg/kg/day); and CI-AKI + atorvastatin group (20 mg/kg/day). CI-AKI was induced by dehydration for 72 hours, followed by furosemide intramuscular injection 20 minutes before low-osmolar contrast media (CM) intravenous injection. Statins were administered by oral gavage once daily for 3 consecutive days before CM injection and once 4 hours after CM injection. Rats were sacrificed 24 hours after CM injection, and renal function, kidney histopathology, nitric oxide (NO) metabolites, and markers of oxidative stress, inflammation, and apoptosis were evaluated. The results showed that atorvastatin and rosuvastatin but not simvastatin ameliorated CM-induced serum creatinine elevation and histopathological alterations. Atorvastatin and rosuvastatin showed similar effectiveness against CM-induced oxidative stress, but simvastatin was less effective. Atorvastatin was most effective against NO system dysfunction and cell apoptosis, whereas rosuvastatin was most effective against inflammation. Our findings indicate that statins exhibit differential effects in preventing CI-AKI when given at equivalent lipid-lowering doses.

## 1. Introduction

Contrast-induced acute kidney injury (CI-AKI), an important adverse effect of iodinated contrast media (CM), accounts for one-third of hospital-acquired AKI cases [1, 2]. Patients with CI-AKI have a higher risk of in-hospital adverse events, prolonged hospital stay, and long-term mortality compared with those without CI-AKI [3]. Therefore, preventive strategies for CI-AKI are urgently needed.

Many studies have reported the cholesterol-lowering effects of statins [4, 5], which exert pleiotropic effects including renoprotection [6]. Several studies as well as most recent meta-analysis have reported that pretreatment with statins prior to CM exposure significantly decreased the incidence of CI-AKI [7–9]; however, other studies have reported conflicting results [10–14]. Toso et al.'s study indicated that statin therapy does not decrease the incidence of CI-AKI and the long-term kidney function loss in addition to standard intravenous hydration and oral N-acetylcysteine [12]. Another recent systematic review indicated that statin therapy might not further reduce the risk of CI-AKI in patients with chronic kidney disease [14]. One potential factor contributing to this discrepancy is variability in the pleiotropic effects of different statins. Chemical properties vary among different statins, which influences the solubility and drug distribution, as well as the bioavailability and pleiotropic effects. We hypothesized that the magnitude of CI-AKI prevention differs between statins for this reason [15]. Therefore, the aim of this study was to compare renoprotective effects of widely prescribed statins (atorvastatin, rosuvastatin, and simvastatin) in a rat model of CI-AKI.

## 2. Materials and Methods

### 2.1. Animals

Male Sprague-Dawley rats (230–250 g) were purchased from SLAC Laboratory Animal Co., Ltd. (Shanghai, China). The rats were kept in individual cages under controlled conditions at 20°C–24°C on a 12 : 12-hour light/dark cycle and had free access to tap water and a standard laboratory diet. The rats were acclimatized for 7 days prior to the start of study. All experimental protocols were approved by the Committee on the Ethics of Animal Experiments of the School of Medicine, Shanghai Jiao Tong University (Permit number RJ-20160820) and were in compliance with the Guide for the Care and Use of Laboratory Animals by the National Academy Press.

### 2.2. Induction of CI-AKI and Experimental Treatment

CI-AKI was induced by using a previously reported method [16]. In brief, rats were deprived of water for 72 hours and then given 10 mg/kg furosemide (Harvest Pharmaceutical Co., Shanghai, China) by intramuscular injection. After 20 minutes, a nonionic, low-osmolar CM (Omnipaque, 350 mg I/mL; GE Healthcare, Shanghai, China) was administered by intravenous injection (10 mL/kg) via the tail vein over the course of 5 minutes.

Forty-five rats were randomly allocated to the following five groups (*n* = 9 per group): (1) control group (dehydration + furosemide, without CM administration); (2) CI-AKI group; (3) CI-AKI + rosuvastatin group (10 mg/kg, AstraZeneca Pharmaceutical Co., Ltd., UK); (4) CI-AKI + simvastatin group (80 mg/kg, Merck & Co., Inc., USA); and (5) CI-AKI + atorvastatin group (20 mg/kg, Pfizer Pharmaceuticals Ltd., USA). Baseline blood samples were collected from the jugular vein under light ether anesthesia at the end of acclimatization period, and the serum was separated from the whole blood. Statins were administered by oral gavage once daily for 3 consecutive days prior to CM injection and once at 4 hours after CM injection. The rats were allowed to recover for 24 hours after the CM injection and then sacrificed by light ethyl ether anesthesia. The final blood samples were collected through the jugular vein. After harvesting the kidneys, the left kidney was stored at −80°C for biochemical analysis, and the right kidney was fixed in 10% formalin for histopathological evaluation.

### 2.3. Evaluation of Renal Function and Definition of AKI

Serum creatinine (SCr) concentration was determined using an automatic biochemical analyzer (Hitachi 7600, Japan) at the central clinical laboratory of Ren Ji Hospital. AKI was defined by a relative increase in SCr ≥ 25% over baseline [16].

### 2.4. Histopathological Analysis of Kidney Tissues

Kidney tissue was fixed in 10% neutral buffered formalin, embedded in paraffin, cut into 3 *μ*m sections, and stained with hematoxylin and eosin (H&E). Histopathological analysis was performed in a blinded manner using a light microscope (Leica DM2500, Leica Microsystems, Wetzlar, Germany). Ten high-magnification (×200) fields of the cortex and outer stripe of the outer medulla were randomly selected for semiquantitative analysis.

The renal lesions were graded according to Yamasowa et al. [17]. Tubular necrosis and proteinaceous casts were graded as follows: 0 = no damage, 1 = mild (unicellular, patchy isolated damage), 2 = moderate (<25% damage), 3 = severe (25%–50% damage), or 4 = very severe (>50% damage). Medullary congestion was graded as follows: 0 = no congestion, 1 = mild (vascular congestion with identification of erythrocytes by ×400 magnification), 2 = moderate (vascular congestion with identification of erythrocytes by ×200 magnification), 3 = severe (vascular congestion with identification of erythrocytes by ×100 magnification), or 4 = very severe (vascular congestion with identification of erythrocytes by ×40 magnification).

### 2.5. Assessment of Oxidative Stress

Thiobarbituric acid reactive substances (TBARS) in kidney tissue and malondialdehyde (MDA) level in serum were assessed as indicators of lipid peroxidation. Kidney TBARS level was measured according to Sözmen et al. [18]. Renal homogenates were incubated with thiobarbituric acid solution for 40 minutes at 95°C. Absorbance was measured at 532 nm, and 1,1,3,3-tetraethoxypropane was used to construct a calibration curve. Serum MDA levels were measured according to the method of Ohkawa et al. [19], and absorbance was determined with the method used for TBARS. Serum thiol, a marker of protein oxidation, was measured according to the method of Hu et al. [20]. Absorbance was measured at 412 nm, and glutathione was used to construct a calibration curve.

### 2.6. Real-Time PCR

Relative mRNA levels of interleukin-6 (IL-6), monocyte chemotactic protein-1 (MCP-1), and tumor necrosis factor-*α* (TNF-*α*) were determined by real-time polymerase chain reaction (PCR). Total RNA was extracted from the kidney tissues using TRIzol reagent (Invitrogen, Carlsbad, CA), according to the manufacturer's instructions. PCR amplification was performed using SYBR Green dye and the LightCycler® 480 Real-Time PCR System (Roche Applied Science, Penzberg, Germany) using the following primers: *β*-actin forward: 5′-GGCATCGTCACCAACTGGGAC-3′, reverse: 5′-CGATTTCCCGCTCGGCCGTGG-3′; IL-6 forward: 5′-TCTTGGGACTGATGTTGTTG-3′, reverse: 5′-TAAGCCTCCGACTTGTGAA-3′; MCP-1 forward: 5′-TGAGTCGGCTGGAGAACTACAAG-3′, reverse: 5′-AGGTGCTGAAGTCCTTAGGGTTG-3′; and TNF-*α* forward: 5′-CACCACGCTCTTCTGTCTACTG-3′, reverse: 5′-GCTTGG TGGTTTGCTACGAC-3′. Relative mRNA level was calculated using the 2^−ΔΔCT^ method and normalized to *β*-actin expression.

### 2.7. Total Nitrite/Nitrate Levels

Nitrite and nitrate levels were determined by using the Griess reaction [21]. Nitrite reacted with sulfanilamide and N-(1-naphthyl)ethylenediamine, and absorbance was measured at 540 nm. Nitrate was first reduced to nitrite, and absorbance was measured at 340 nm. Sodium nitrite and nitrate were used to construct calibration curves.

### 2.8. Terminal Deoxynucleotidyl Transferase dUTP Nick-End Labeling (TUNEL) Assay

To detect apoptotic DNA fragmentation, TUNEL staining was performed using the In Situ Cell Death Detection Kit (Roche Diagnostics, Madison, WI, USA) according to the manufacturer's instructions. Digital images were acquired by confocal microscopy (LSM 710, Zeiss, Oberkochen, Germany). The integrated optical density of the TUNEL-stained tubular cells was assessed using Image Pro Plus software (Media Cybernetics, Rockville, MD, USA) in a blinded manner. Results are expressed as percentage of TUNEL-positive cells.

### 2.9. Western Blotting

Kidney tissue was homogenized in lysis buffer (Roche Diagnostics, Madison, WI, USA) and then centrifuged at 12,000*g* for 15 minutes at 4°C. Protein in the supernatants was quantified using the BCA protein assay reagent (Pierce, Waltham, MA, USA), separated by 12% SDS-PAGE, and transferred to nitrocellulose membranes. The membranes were incubated with primary antibodies against Bax (1 : 1000, Cell Signaling Technology, Danvers, MA, USA), Bcl-2 (1 : 1000, Cell Signaling Technology, Danvers, MA, USA), and *β*-actin (1 : 5000, Abcam, Cambridge, UK), followed by incubation with horseradish peroxidase-conjugated secondary antibodies (1 : 5000, Bio-Rad Laboratories, Hercules, USA). Proteins were detected using ECL Western blotting detection reagent (Pierce, Rockford, IL, USA) and quantified using the ImageQuant LAS 4000 mini system (GE Healthcare Bio-Sciences AB, Uppsala, Sweden).

### 2.10. Statistical Analysis

The group sample size was estimated by G^*∗*^Power 3.1 software (Heinrich Heine, Germany) based on the result of a preliminary study of twenty rats (*n* = 4 per group) [22]. To achieve 95% power with the statistically significant level at 0.05 for group difference in SCr at 24 hours after CM injection among five groups, nine rats were required in each group. Results are expressed as mean ± standard error of the mean (SEM). The paired Student *t*-test was used for within-subject comparisons. Comparisons among groups were made using one-way analysis of variance, followed by Tukey's test for multiple comparisons. *P* < 0.05 was considered significant. Analyses were performed using SPSS software version 18.0 (SPSS, Chicago, IL, USA).

## 3. Results

### 3.1. Effects of Statins on Renal Function in CI-AKI Rats

Renal function parameters are shown in Table 1. Our results showed that no significant differences existed in baseline SCr levels among five groups. SCr level at 24 hours after CM injection was increased by 31% in the CI-AKI group compared with the control group (*P* < 0.01). Treatment with rosuvastatin decreased SCr levels by 14% (*P* < 0.05), and treatment with atorvastatin decreased SCr levels by 22% (*P* < 0.01) compared with the CI-AKI group. In contrast, treatment with simvastatin did not significantly decrease SCr levels in rats with CI-AKI. AKI rates of each group were also presented in Table 1.

### 3.2. Effects of Statins on Kidney Histopathological Alterations in CI-AKI Rats

**Figure 1:**
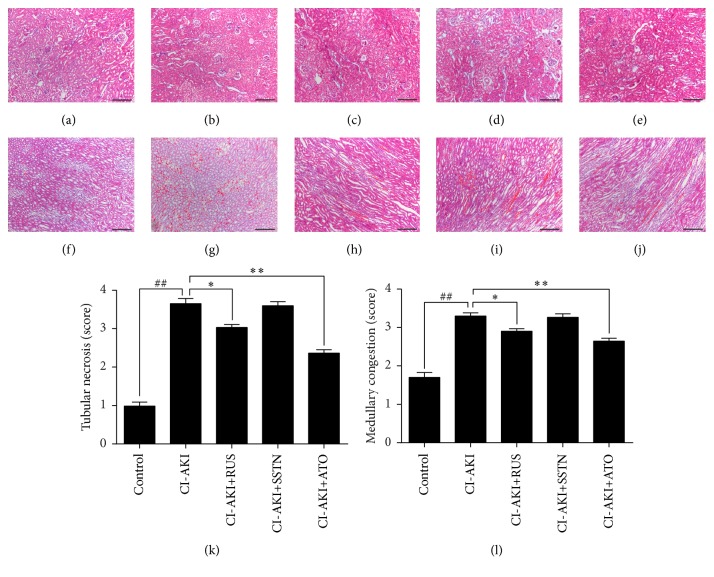
Histopathological evaluation of kidney tissues in a rat model of CI-AKI with or without statin treatment. Representative photomicrographs show H&E-stained kidney sections (a–j) and semiquantitative analysis of tubular necrosis and medullary congestion (k, l). Control group (a, f); CI-AKI group (b, g); CI-AKI + RUS (c, h); CI-AKI + SSTN (d, i); and CI-AKI + ATO (e, j). Original magnification: ×100. Calibration bar = 200 *μ*m. Results are expressed as mean ± SEM (*n* = 9). ^##^*P* < 0.01 versus control group; ^*∗∗*^*P* < 0.01, ^*∗*^*P* < 0.05 versus CI-AKI group. ATO: atorvastatin; CI-AKI: contrast-induced acute kidney injury; H&E: hematoxylin and eosin; RUS: rosuvastatin; SSTN: simvastatin.

In the kidney sections of rats, H&E staining showed subtle histopathological changes in control group (Tubular necrosis score: 0.98 ± 0.10, Figure 1(a); medullary congestion score: 1.70 ± 0.13, Figure 1(f)), but severe tubular necrosis (score: 3.65 ± 0.14, Figure 1(b)) and medullary congestion (score: 3.30 ± 0.08, Figure 1(g)) in CI-AKI group. However, histopathological alterations were attenuated in CI-AKI rats treated with rosuvastatin (Figures 1(c) and 1(h)) or atorvastatin (Figures 1(e) and 1(j)). Compared with untreated CI-AKI, treatment with rosuvastatin lowered the tubular necrosis by 20% (*P* < 0.05) and medullary congestion by 12% (*P* < 0.05), and treatment with atorvastatin lowered the tubular necrosis by 36% (*P* < 0.01) and medullary congestion by 20% (*P* < 0.01) (Figures 1(k) and 1(l)). In contrast, treatment with simvastatin did not attenuate tubular necrosis or medullary congestion. Overall, the histopathological alterations in rats treated with the three statins were consistent with changes in renal function.

### 3.3. Effects of Statins on Oxidative Stress in CI-AKI Rats

**Figure 2:**
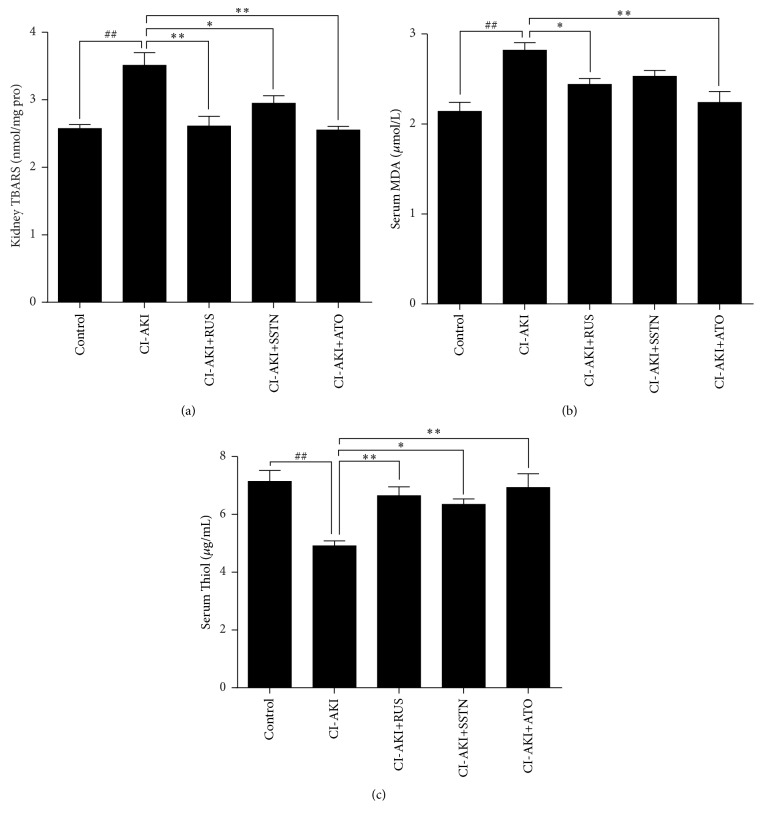
Effects of statins on oxidative stress in a rat model of CI-AKI. Oxidative stress was evaluated by changes in the levels of (a) hepatic TBARS, (b) serum MDA, and (c) serum thiol. Results are expressed as mean ± SEM (*n* = 9). ^##^*P* < 0.01 versus control group; ^*∗∗*^*P* < 0.01, ^*∗*^*P* < 0.05 versus CI-AKI group. ATO: atorvastatin; CI-AKI: contrast-induced acute kidney injury; MDA: malondialdehyde; RUS: rosuvastatin; SSTN: simvastatin; TBARS: thiobarbituric acid reactive substances.

Renal TBARS, serum MDA, and serum thiol levels were assessed as markers of oxidative stress (Figure 2). Our results showed that renal TBARS levels increased in the CI-AKI group by 37% (*P* < 0.01) compared to the control group, and treatment with rosuvastatin, simvastatin, and atorvastatin decreased the TBARS level by 25% (*P* < 0.01), 16% (*P* < 0.05), and 27% (*P* < 0.01), respectively, compared with the CI-AKI group. The serum MDA level was increased in the CI-AKI group by 32% (*P* < 0.01) compared to the control group, and treatment with rosuvastatin and atorvastatin decreased serum MDA by 13% (*P* < 0.05) and 21% (*P* < 0.01), respectively. Treatment with simvastatin appeared to decrease the MDA level (10%) compared to the CI-AKI group, but this difference was not significant. The serum thiol level was decreased in the CI-AKI group by 31% (*P* < 0.01) compared to the control group, and treatment with rosuvastatin, simvastatin, and atorvastatin increased serum thiol by 35% (*P* < 0.01), 29% (*P* < 0.05), and 41% (*P* < 0.01), respectively.

### 3.4. Effects of Statins on Renal Inflammation in CI-AKI Rats

**Figure 3:**
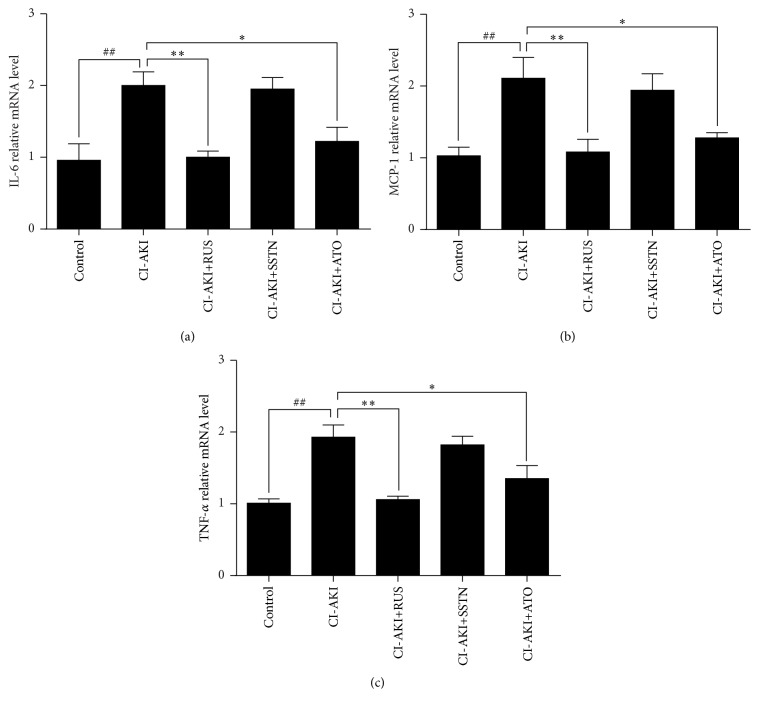
Effects of statins on renal inflammation in a rat model of CI-AKI. Relative mRNA levels of (a) IL-6, (b) MCP-1, and (c) TNF-*α* were assessed in kidney tissue, using tissue, as an internal control. Results are expressed as mean ± SEM of three independent experiments. ^##^*P* < 0.01 versus control group; ^*∗∗*^*P* < 0.01, ^*∗*^*P* < 0.05 versus CI-AKI group. ATO: atorvastatin; CI-AKI: contrast-induced acute kidney injury; IL-6: interleukin-6; MCP: monocyte chemotactic protein-1; RUS: rosuvastatin; SSTN: simvastatin; TNF-*α*: tumor necrosis factor-*α*.

IL-6, MCP-1, and TNF-*α* were assessed as markers of inflammation (Figure 3). Relative mRNA levels of IL-6, MCP-1, and TNF-*α* in kidney tissues were increased in the CI-AKI group by 108% (*P* < 0.01), 104% (*P* < 0.01), and 91% (*P* < 0.01), respectively, compared with the control group. Treatment with rosuvastatin decreased IL-6, MCP-1, and TNF-*α* mRNA levels by 50% (*P* < 0.01), 49% (*P* < 0.01), and 45% (*P* < 0.01), respectively, compared with the CI-AKI group. Treatment with atorvastatin decreased IL-6, MCP-1, and TNF-*α* mRNA levels by 39% (*P* < 0.05), 39% (*P* < 0.05), and 30% (*P* < 0.01), respectively, compared with the CI-AKI group. In contrast, treatment with simvastatin did not significantly decrease mRNA levels of these inflammatory markers.

### 3.5. Effects of Statins on Nitric Oxide Metabolism in the Kidneys of CI-AKI Rats

As shown in Table 2, levels of NO metabolites (nitrite and nitrate) were decreased by 18% in CI-AKI rats compared with the control group (*P* < 0.01). Atorvastatin treatment blocked this effect, with atorvastatin-treated CI-AKI rats showing higher total NO metabolite levels than the untreated CI-AKI rats (*P* < 0.05). In contrast, rosuvastatin and simvastatin did not significantly change nitrite/nitrate levels.

### 3.6. Effects of Statins on Apoptosis in the Kidneys of CI-AKI Rats

**Figure 4:**
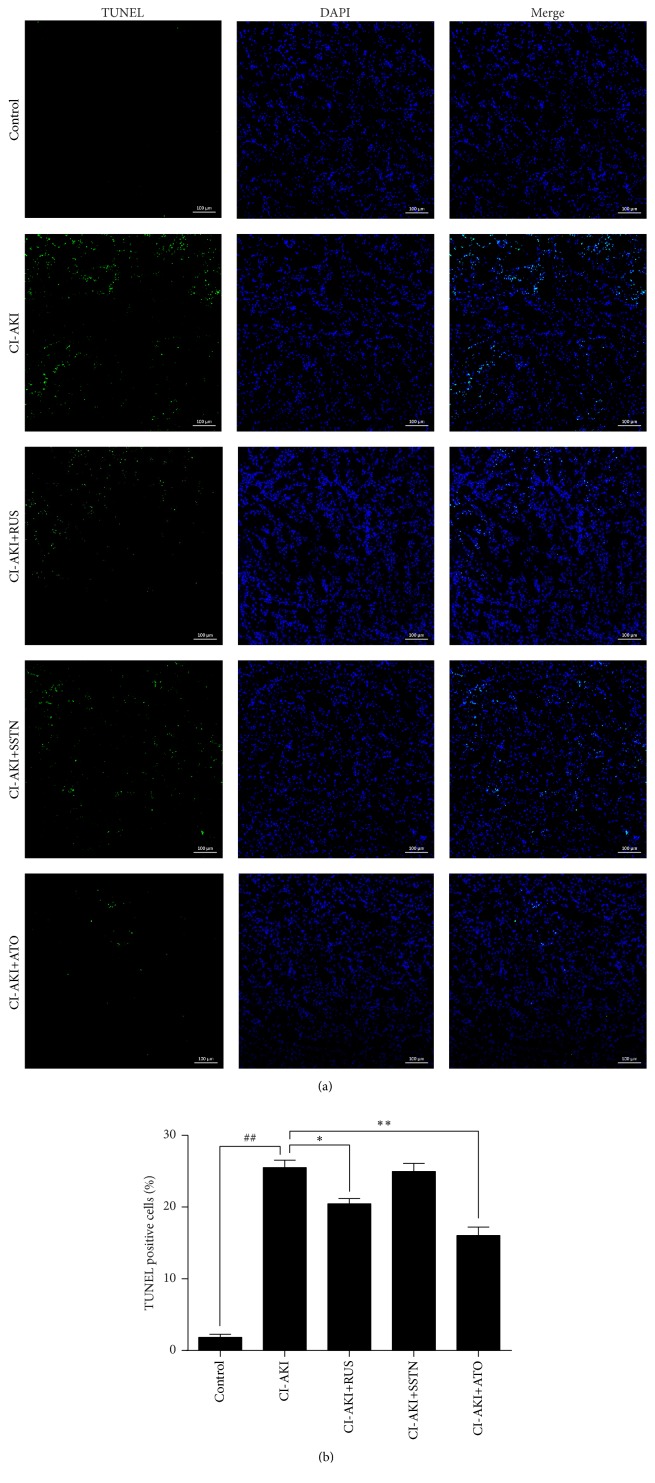
Effects of statins on apoptotic cell death in rat kidney tissues, as assessed by TUNEL staining. (a) Representative photomicrographs of kidney tissue sections. DNA fragmentation was visualized by TUNEL staining (green). Nuclei were stained with DAPI (blue). (b) Quantitative analysis of apoptotic cell death (percentage of TUNEL-positive cells). Original magnification: ×100. Calibration bar = 100 *μ*m. Results are expressed as mean ± SEM (*n* = 9). ^##^*P* < 0.01 versus control group; ^*∗∗*^*P* < 0.01, ^*∗*^*P* < 0.05 versus CI-AKI group. ATO: atorvastatin; CI-AKI: contrast-induced acute kidney injury; DAPI: 4′,6-diamino-2-phenylindole; RUS: rosuvastatin; SSTN: simvastatin; TUNEL: terminal deoxynucleotidyl transferase dUTP nick-end labeling.

**Figure 5:**
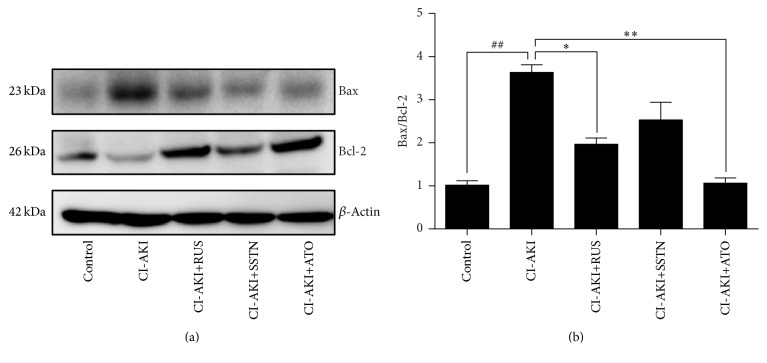
Effects of statins on expression of Bax and Bcl-2 in rat kidney tissues. (a) Levels of Bax and Bcl-2 were determined by Western blot analysis, using *β*-actin as an internal control. (b) Quantification of Bax/Bcl-2 ratio. Results are expressed as mean ± SEM of three independent experiments. ^##^*P* < 0.01 versus control group; ^*∗∗*^*P* < 0.01, ^*∗*^*P* < 0.05 versus CI-AKI group. ATO: atorvastatin; CI-AKI: contrast-induced acute kidney injury; RUS: rosuvastatin; SSTN: simvastatin.

Assessment of apoptotic DNA fragmentation showed an approximately 13-fold increase in the number of TUNEL-positive cells in kidney sections of CI-AKI rats (*P* < 0.01) compared with the control group (Figure 4). Treatment with rosuvastatin and atorvastatin decreased the number of TUNEL-positive cells by 20% (*P* < 0.05) and 37% (*P* < 0.01), respectively, compared with the CI-AKI group, whereas simvastatin had no effect. Bax/Bcl-2 ratios were consistent with the results of TUNEL staining in the five groups (Figure 5).

## 4. Discussion

Recent studies have evaluated the effects of statins in preventing CI-AKI, but the results have been conflicting [4, 11, 14]. Statins are commonly prescribed lipid-lowering agents but have multiple secondary effects [6, 23, 24], and the degree of these pleiotropic effects appears to vary between statins [15]. This variation may be an important confounding factor in studies evaluating the use of statins in CI-AKI.

To address this issue, two clinical trials compared the efficacy of atorvastatin versus rosuvastatin in CI-AKI prevention. The ROSA-cIN trial was a randomized controlled trial that enrolled 192 patients with acute ST segment elevation myocardial infarction who received atorvastatin (80 mg) or rosuvastatin (40 mg) prior to primary percutaneous coronary intervention [25]. The other trial was an observational study of 1078 patients with chronic kidney disease who received atorvastatin (20 mg daily, *n* = 805) or rosuvastatin (10 mg daily, *n* = 273) 2-3 days prior to CM exposure [26]. Results of these studies did not demonstrate the superiority of either statin. However, these two studies may have lacked the power to detect significant differences in treatment effects because of inadequate sample size and potential sample selection bias. Moreover, using change in SCr as a surrogate endpoint may underestimate the real effects of statins because SCr is not sensitive enough to detect renal parenchymal injury [27].

To compare the ability of various statins to prevent CI-AKI, we used a rat model of CI-AKI. Inducing dehydration and pretreating with furosemide simulates the renal hypoperfusion, elevated SCr levels, and histopathologic alterations in kidney tissue observed in patients [16]; therefore, no other pharmacological or surgical procedures are needed. We chose the dose of rosuvastatin based on a study by Deng et al. [28], in which rosuvastatin (10 mg/kg daily) attenuated the severity of CM-induced kidney injury in rats. We then chose doses of atorvastatin and simvastatin with equivalent lipid-lowering effects. The major findings of our study were as follows: (1) atorvastatin and rosuvastatin but not simvastatin exerted renoprotective effects in CI-AKI rats; (2) treatment with atorvastatin achieved better results than rosuvastatin with regard to renal function parameters and histopathological alterations; (3) atorvastatin and rosuvastatin were similarly effective against CM-induced oxidative stress, but simvastatin was less effective; (4) atorvastatin was most effective against CM-induced NO system dysfunction and apoptosis; and (5) rosuvastatin was most effective against CM-induced inflammation. Our results indicate that statins exhibit differential effects in CI-AKI prevention when given at equivalent lipid-lowering doses. These results must be confirmed by randomized controlled trials with larger sample sizes using more sensitive markers of renal parenchymal injury, such as neutrophil gelatinase-associated lipocalin or microRNAs [29, 30].

Several study limitations should be mentioned. First, dose and duration response were not evaluated in this study. Thus, future studies are needed to determine the optimal dose and duration of statin treatment to prevent CI-AKI. Second, the precise mechanisms underlying the antioxidant, anti-inflammatory, and antiapoptotic effects of these statins were not investigated. Finally, reasons for the differential effects of the statins evaluated in this study are unclear. However, lipophilicity of the statin may be a potential explanation. Lipophilic statins such as atorvastatin enter cells by passive diffusion and distribute more widely in organs compared with hydrophilic statins such as rosuvastatin [31]. Therefore, atorvastatin may be more “renophilic” than rosuvastatin. However, this property would not explain why simvastatin, another lipophilic statin, showed lower efficacy than atorvastatin in our study. Thus, studies investigating the relationship between the chemical properties, bioavailability, and pharmacokinetic characteristics of statins and their renoprotective effects are needed.

## 5. Conclusions

In conclusion, we show here for the first time that statins produce variable renoprotective effects in a rat model of CI-AKI. Atorvastatin and rosuvastatin ameliorated CI-AKI in rats and showed similar effectiveness in decreasing oxidative stress. In contrast, simvastatin was not effective against CI-AKI. Atorvastatin was most effective in attenuating nitric oxide system dysfunction and cell apoptosis, whereas rosuvastatin was most effective in decreasing inflammation.

## Figures and Tables

**Table 1:** Effects of statins on renal function, in rats with contrast-induced acute kidney injury (CI-AKI).

	Control (*n* = 9)	CI-AKI (*n* = 9)	CI-AKI + RUS (*n* = 9)	CI-AKI + SSTN (*n* = 9)	CI-AKI + ATO (*n* = 9)
SCr at baseline (*μ*mol/L)	22.89 ± 1.15	22.70 ± 1.11	23.08 ± 1.17	23.50 ± 1.02	22.67 ± 0.93
SCr at 24 h after CM injection (*μ*mol/L)	23.89 ± 0.56	31.30 ± 0.79^##,††^	26.92 ± 1.19^*∗*,†^	30.17 ± 1.09^††^	24.33 ± 1.13^*∗∗*^
Change in SCr above baseline (%)	2.78 ± 3.16	32.11 ± 4.53^##^	11.64 ± 5.14^*∗*^	24.10 ± 4.35	6.44 ± 5.53^*∗∗*^
AKI rate	0% (0/9)	88.89% (8/9)	44.44% (4/9)	66.67% (6/9)	22.22% (2/9)

Results are expressed as mean ± SEM. ^##^*P* < 0.01 versus control group, ^*∗*^*P* < 0.05 versus CI-AKI group, ^*∗∗*^*P* < 0.01 versus CI-AKI group, ^†^*P* < 0.05 versus SCr at baseline, and ^††^*P* < 0.01 versus SCr at baseline.

ATO: atorvastatin; CM: contrast media; RUS: rosuvastatin; SCr: serum creatinine; SSTN: simvastatin.

**Table 2:** Effects of statins on nitric oxide metabolites in kidney tissue in a rat model of contrast-induced acute kidney injury (CI-AKI).

	Control (*n* = 9)	CI-AKI (*n* = 9)	CI-AKI + RUS (*n* = 9)	CI-AKI + SSTN (*n* = 9)	CI-AKI + ATO (*n* = 9)
Total nitrite/nitrate (nmol/mg protein)	5.91 ± 0.60	4.85 ± 0.16^##^	5.09 ± 0.45	4.98 ± 0.50	6.40 ± 0.17^*∗*^
Nitrite (nmol/mg protein)	1.53 ± 0.15	1.26 ± 0.08	1.39 ± 0.06	1.37 ± 0.09	1.77 ± 0.18
Nitrate (nmol/mg protein)	4.38 ± 0.54	3.60 ± 0.10	3.69 ± 0.49	3.60 ± 0.50	4.63 ± 0.22

Results are expressed as mean ± SEM. ^##^*P* < 0.01 versus control group; ^*∗*^*P* < 0.05 versus CI-AKI group.

ATO: atorvastatin; RUS: rosuvastatin; SSTN: simvastatin.
